# Heavy metals effect on breast cancer progression

**DOI:** 10.1186/s12995-017-0178-1

**Published:** 2017-11-28

**Authors:** А. Romaniuk, M. Lyndin, V. Sikora, Y. Lyndina, S. Romaniuk, K. Sikora

**Affiliations:** 1 Department of pathology, Sumy State University, st. Privokzalnaya, 31, Sumy, Postal code 40022 Ukraine; 2 Department of normal anatomy, Sumy State University, Sumy, Ukraine; 3 Cardiology department of Sumy regional hospital, Sumy, Ukraine; 4 Sumy Regional Clinical Perinatal Center, Sumy, Ukraine

**Keywords:** Breast cancer, Heavy metals, DNA methylation, Receptors

## Abstract

**Background:**

Breast cancer is the most frequent localization of malignant process in American women and women of European countries. To date it is not possible to control the morbidity growth due to lack of effective ways of primary prevention. Comparing the incidence of breast cancer in developed countries with the countries of Asia and Africa, there is the fact of population predominance lesion in more urbanized countries. This suggests that the environment along with other factors, occupies a significant place in the initiation and progression of breast neoplasia. The impressive rates of industrial development led to the pollution of soil, surface water and, as a consequence, food by heavy metal salts.

The purposes of this paper are as follows: the chemical composition determination of neoplastic breast tissue, evaluation of the DNA methylation level, study of prognostic-important receptors expression in the breast cancer cells, establishing linkages between all the derived indicators.

**Methods:**

In our study we used the following methods: studying of the chemical composition of breast cancer tissue by atomic absorption spectrophotometry and energy-dispersion spectrometer; іmmunohistochemical study of ER, PR, HER2/neu, p53, Ki-67, E-cadherin and MGMT receptors; DNA extraction and investigation by oscillating infrared spectroscopy method.

**Results:**

The total amount of heavy metals in breast cancer tissue ranged from 51.21 × 10^−3^ to 84.86 × 10^−3^ μg/kg. We have got the following results: the growth of heavy metals in neoplastic tissue is accompanied with the increase of HER2/neu, p53, Ki-67, MGMT expression and decrease of ER and PR expression. The increment of pathological DNA methylation is accompanied with the increasing amount of heavy metals in tumor tissue.

**Conclusions:**

Heavy metals through different pathogenetic links stimulate the progression of breast cancer and reduce its sensitivity to treatment. DNA of tumor tissue has a different level of methylation which changes with the amount of heavy metals in cancer cells. This is displayed on the synthesis of prognostically important receptors in neoplastic tissue.

## Background

Breast cancer (ВС) is the most frequent localization of malignant process in American women and women of European countries [1]. In Ukraine, this pathology is found in 16,000 women each year (the incidence is 64.5 per 100,000 women) [2]. Nowadays it isn’t possible to control the growth of morbidity due to lack of effective ways of primary prevention. Comparing the incidence of ВС in developed countries with the countries of Asia and Africa, unfortunately, there is the fact of predominance lesion of population in more urbanized countries [3]. Thus, this suggests that the environment along with other factors, occupies a significant place in both the initiation and progression of breast neoplasia [4]. The impressive rates of industrial development led to the pollution of soil, surface water and, as a consequence, food by heavy metal salts.

During investigation of the environment in Ukraine it was found that the amount of iron, chromium, copper, nickel, lead and zinc salts in the ground were increased [5].

It is known that the carcinogenic effect of heavy metals (HM) is realized through the mechanisms of DNA structure breach and suppression of antioxidant protection [6, 7]. Furthermore, there is information about the possibility of heavy metals influence on the expression of prognostic-important receptors in BC tissue [8, 9]. Previously, it was found that breast tissue (unaltered and affected by tumor process) can accumulate heavy metals that influence on the DNA fragmentation level and tumor cells survival [10].

It is generally known that the most important receptors in BC tissue are the estrogen receptor (ER), progesterone receptor (PR), human epidermal growth factor receptor 2 (HER2/neu), Ki-67 and p53. Moreover, ER and PR status of BC is recognized as one of the most powerful prognostic markers. If the tumor belongs to steroid-positive variant, it is managed and controlled by anti-estrogen drugs [11]. Contrary to this, the expression of HER2/neu indicates the tumor resistance to chemotherapy, including tamoxifen, aggressiveness of cancer [12]. The degree of differentiation and malignancy of BC is largely determined by proliferative activity of tumor cells. Study of Ki-67 protein that is expressed in all cells that came from G_0_-phase allows to determine “hidden” proliferative potential of neoplasia and to evaluate its malignancy extent [13]. Pathological p53 presents in 18%-25% of BC cases. Functional inactivation of wild type (wt) p53 and synthesis of mutant type (mt) p53 are associated with early recurrences, poorer course and resistance to chemotherapy [14].

DNA methylation, the accession of methyl groups to the nitrogenous bases, is a modification of DNA without changing its nucleotide sequence [15]. “Silence” of tumor suppressor genes by DNA methylation is a powerful molecular mechanism by which DNA methylation can initiate cancer. It also substantiates therapy that aims to inhibit DNA methylation, and reactivation of blocked tumor suppressor genes [16]. It has been described a significant correlation between DNA methylation and expression of BC key genes such as MYC, TERT, TP63 and others [17]. However, hypomethylation is no less important because the critical genes of growth and metastasis are hypomethylated in cancer tissue [18].

O6-methyguanine-DNA methyltransferase (MGMT), with its gene being located on the chromosome 10, eliminates DNA damage by alkylating agents, removing their cytotoxic effect [19]. Afferent protein sequence is combined with damaged nucleic acid and without changing DNA structure takes alkyl residue [20]. Nevertheless, inactivated MGMT may enter into relationship with receptors for steroid hormones, inhibiting their function, thereby blocking the signal transmission for cell division [21]. There is information about inhibitory effect of heavy metal salts on MGMT activity. It has been shown that the expression of MGMT in tumor cells can be increased, demonstrating BC resistance against chemotherapy [22], and can be decreased (due to methylation of MGMT promoter), providing the possibility of “cancer” cells enter into apoptosis [23]. MGMT-positive status of the tissue is correlated with the identification of luminal molecular types of BC and low cell proliferative activity [24]. Considering this, it was proposed to examine the expression of MGMT as a separate prognostic factor of breast malignancy [23].

In summary we can say that nowadays the question remains open concerning the relationship between the tumor chemical composition, DNA methylation and expression of prognostic receptors of BC tissue.

The **objectives** of this paper are as follows: the chemical composition determination of neoplastic breast tissue, evaluation of the DNA methylation level, study of prognostic-important receptors expression in the BC cells, establishing linkages between all the derived indicators.

## Methods

The tissue (40 samples of BC tissue and 20 samples of intact breast tissue) received during the postoperative biopsy was kept in the refrigerator. It became the subject of the further investigation after the establishing of the histological diagnosis. The diagnosis of invasive ductal carcinoma (IDC) with varying of malignancy degree (by Scarff-Bloom-Richardson) was found after the standard hematoxylin eosin staining of prepared slices.

### Studying of the chemical composition of BC tissue

Tissue samples for atomic absorption spectrophotometry were weighed with an accuracy of 0.001 g. Then they were burned in a muffle furnace at a temperature 450 °C for removing the organic matrix. The resulting ash was dissolved in a mixture of hydrochloric (2 ml) and nitric (1 ml) acids. The resulting solution was analyzed by C115 − 01 spectrophotometer with flame and electrothermal atomiser. Each microelement (increased amount of which is inherent for the Ukraine) content was measured at a specific wavelength: Zn – 213.9 nm, Cu – 324.7 nm, Pb – 283.3 nm, Cr – 357.9 nm, Fe – 248.3 nm and Ni – 240.7 nm. Measurements and calculations were performed by «AAS-SPECTR» program.

Paraffin sections (of 5 μm thickness) for energy dispersive spectrometry were subjected to dewaxing and applied on spectral pure graphite rods. Chemical composition was studied by means of the scanning electron microscope with energy-dispersion spectrometer [25]. Digital images of the specimen and the indicators of microelements content were identified by Magеllanes and VCU software.

### Immunohistochemical study

The material for the immunohistochemical reaction was fixed in 10% neutral formalin for 24 h, after that paraffin blocks were made of it. Then sections of 3-4 μm thickness were made and they were subjected to the standard process of dehydration in xylol and alcohols of rising concentration. Immunohistochemical reaction consisted of 2 stages. During the first stage the incubation with primary rabbit antibodies («Thermo Fisher Scientific», USA) in different dilutions in tissue sections took place (Table 1). Then we conducted the receptor visualization using detection systems «UltraVision Quanto Detection System HRP DAB Chromogen» («Thermo Fisher Scientific», USA). We visualized the cell structural components using diaminobenzidine, which painted them in a brown color. We have performed immunohistochemical reaction with E-cadherin for the purpose of the differential diagnosis between high degree malignant invasive lobular carcinoma and IDC. The positive reaction took place only in IDC.

**Table 1:** Antibody for immunohistochemical reaction («Thermo Fisher Scientific», USA)

Antibody	Host	Clone	Dilution	Cellular localization
ERα	Rabbit	SP1	1:200	Nuclear
PR	Rabbit	YR85	1:150	Nuclear
HER2/neu	Rabbit	SP3	1:100	Membrane
p53	Mouse	SP5	1:100	Nuclear
Кі-67	Rabbit	SP6	1:100	Nuclear
MGMT	Mouse	МТ3.1	1:50	Cytoplasmic and nuclear
E-cadherin	Rabbit	67A4	1:100	Membrane

### DNA extraction and investigation

DNA was extracted from the tissue using a lysis buffer which consisted of 30 mM hydroxymethylaminomethane, 10 mM еthylenediamine-tetraacetic acid, 1% sodium dodecyl sulfate and 5 mg proteinase K (pH 8.0). DNA was purified with phenol-chloroform extraction and the following precipitation in absolute ethanol.

DNA methylation level was determined at the Institute for Single Crystals, National Academy of Sciences (Ukraine) using the oscillating infrared spectroscopy method. An obligatory condition was the deprivation of samples of auxiliary substances (absolute ethanol, chloroform, phenol and others) that were used for DNA extraction, and that could overcharge DNA spectrum. Obtained DNA product was triturated with KBr and placed into tablets for infrared spectroscopy on the SpectrumOne spectrometer. Spectrum analysis was performed by OridionVersion8 program.

### Statistical analysis of the results

Mathematical calculations were done using Microsoft Excel 2010 with 12.0.5 Attestat option. We have found such indicators as chi-squared Pearson’s test, Pearson’s correlation coefficient (with statistical significance *p* < 0.05).

## Results

**Fig. 1:**
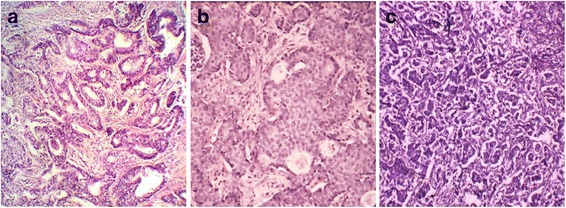
Invasive ductal carcinoma of varying degrees of malignancy by Scarff-Bloom-Richardson. **a** – well-differentiated carcinoma, **b** – moderately differentiated carcinoma, **c** – poorly differentiated carcinoma. Staining with hematoxylin and eosin. Magnification ×100

The invasive ductal carcinoma is characterized by the formation of nests, clusters and trabeculas, though some tumors are characterized by solid growth with a small amount of stroma. Some part of the tumor has clear tubular structures with lumen in the center. The cells are quite variable in structure. The cytoplasm is often broad and eosinophilic. The nuclei are round, monomorphic or with polymorphism and well-visible nucleoli. The mitotic activity varies greatly - from full absence to 10-20 or more in one field of view (Fig. 1).

Using atomic absorption spectrophotometry of IDC tissue it has been found that the content of iron was from 38.46 × 10^-3^ to 69.63 × 10^-3^ μg/kg (average 59.73 × 10^-3^ μg/kg), copper – from 2.8 × 10^-3^ to 9.11 × 10^-3^ μg/kg (average 5.66 × 10^-3^ μg/kg), chromium – from 0 to 6.1 × 10^-3^ μg/kg (average 2.41 × 10^-3^ μg/kg), zinc – from 1.89 × 10^-3^ to 6.4 × 10^-3^ μg/kg (average 4.25 × 10^-3^ μg/kg), lead – from 0 to 0.6 × 10^-3^ μg/kg (average 0.11 × 10^-3^ μg/kg) and nickel – from 0.08 × 10^-3^ to 0.52 × 10^-3^ μg/kg (average 0.3 × 10^-3^ μg/kg). The total amount of HM ranged from 51.21 × 10^-3^ to 84.86 × 10^-3^ μg/kg (average 72.44 × 10^-3^ μg/kg). In near located intact breast tissue these levels were much lower: iron on the average 34,26 × 10^-3^ μg/kg, copper – 2.73 × 10^-3^ μg/kg, chromium – 0.92 × 10^-3^ μg/kg, zinc – 1.67 × 10^-3^ μg/kg, lead – 0.04 × 10^-3^ μg/kg and nickel – 0.1 × 10^-3^ μg/kg (Table 2).

**Table 2:** The content of HM in cancerous and healthy breast tissue (×10^-3^ μg/kg)

	Iron	Copper	Zinc	Lead	Chromium	Nickel	Total
1.	41.36	4.23	3.74	0.16	1.46	0.26	51.21
2.	44.17	3.42	4.17	0.09	0.72	0.34	52.91
3.	38.46	4.8	4.1	0.18	5.1	0.42	53.06
4.	39.65	4.71	5.11	0.14	3.41	0.23	53.25
5.	51.27	5.57	1.89	0.14	1.32	0.28	60.47
6.	52.26	4.32	4.08	0.01	0.65	0.34	61.66
7.	49.38	6.56	3.35	0.15	2.46	0.29	62.19
8.	52.44	4.89	3.29	0	1.78	0.28	62.68
9.	47.81	6.32	3.89	0.07	4.53	0.28	62.9
10.	53.48	5.82	3.23	0.18	1.56	0.32	64.59
11.	52.4	5.61	5.09	0.14	1.78	0.29	65.31
12.	56.54	3.56	3.54	0.01	2.12	0.3	66.07
13.	55.84	6.18	3.65	0	2.11	0.25	68.03
14.	54.63	6.19	5.33	0.16	3.1	0.35	69.76
15.	61.41	5.13	2.93	0.08	0.78	0.26	70.59
16.	60.87	3.65	4.48	0.19	2.23	0.28	71.7
17.	62.62	2.8	4.1	0.09	2.47	0.34	72.42
18.	61.89	3.69	4.44	0.16	2.32	0.34	72.84
19.	60.55	6.21	3.45	0.06	2.51	0.32	73.1
20.	68.39	4.81	2.8	0.09	0	0.16	76.25
21.	64.33	7.25	3.36	0	1.52	0.29	76.75
22.	64.48	5.25	4.88	0.04	2.86	0.36	77.87
23.	65.86	4.23	3.86	0.03	3.62	0.28	77.88
24.	63.81	5.26	5.23	0.11	3.51	0.27	78.19
25.	62.57	8.11	5.69	0.03	1.85	0.35	78.6
26.	63.23	8.66	5.78	0.14	1.69	0.08	79.58
27.	65.59	6.36	4.77	0.19	2.45	0.26	79.62
28.	65.21	7.63	4.11	0	2.56	0.28	79.79
29.	69.63	5.77	2.75	0.14	1.32	0.28	79.89
30.	68.12	5.11	2.84	0.11	3.54	0.28	80
31.	67.54	2.99	5.66	0.08	3.66	0.28	80.21
32.	66.25	7.36	4.28	0.14	2.36	0.32	80.71
33.	68.33	7.68	3.65	0.11	0.92	0.33	81.02
34.	68.87	5.12	4.26	0.08	2.66	0.3	81.29
35.	68.65	5.36	3.96	0	3.15	0.29	81.41
36.	67.2	5.17	6.11	0.12	2.63	0.3	81.53
37.	67.25	6.29	4.85	0.06	3.6	0.25	82.3
38.	66.65	8.62	4.42	0.16	2.24	0.29	82.38
39.	68.11	6.59	6.38	0	1.86	0.35	83.29
40.	62.13	9.11	6.4	0.6	6.1	0.52	84.86
Minimum value	38.46	2.8	1.89	0	0	0.08	51.21
Maximum value	69.63	9.11	6.4	0.6	6.1	0.52	84.86
Average value	59.73	5.66	4.25	0.11	2.41	0.3	72.44
Average value in healthy breast tissue	34.26	2.73	1.67	0.04	0.92	0.1	39.72

**Fig. 2:**
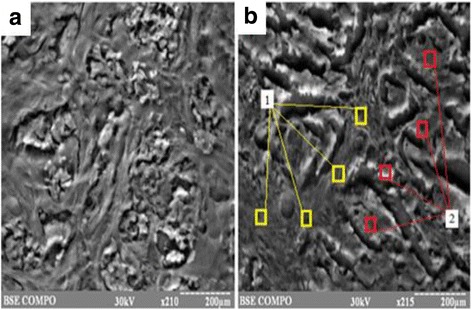
The scanning images of IDC tissue. **a** – local scanning of the sample surface (х210), **b** – focal scanning (х215): 1 – stromal component of tissue, 2 – parenchymal component of tissue

In order to determine the spatial features of HM localization in “cancer” tissue we studied the chemical composition of IDC by energy dispersion method on scanning electron microscope. This process took place in two functional modes: local scanning of the sample surface at different zoom levels and focal scanning, considering trace-element composition of parenchymal and stromal components of BC tissue (Fig. 2). Such macroelements as calcium, phosphorus, potassium, sulfur and sodium dominated in neoplastic tissue. Such heavy metals as zinc, iron, copper, chromium, nickel and lead are encountered in different proportions among other microelements. We have found that HM accumulated more in the parenchymal component of tumor tissue (*p* < 0.05). Comparing the data of two studies on the detection of the chemical composition we’ve got identical results – if the concentration of HM in a gram of tumor was increased then the percentage content also grew that have been obtained using energy-dispersive method of study (p < 0.05). The relationship (*r* = 0.62, *p* < 0.01) between the HM accumulation in the foci of neoplasia and the degree of malignancy of BC has been established.

**Fig. 3:**
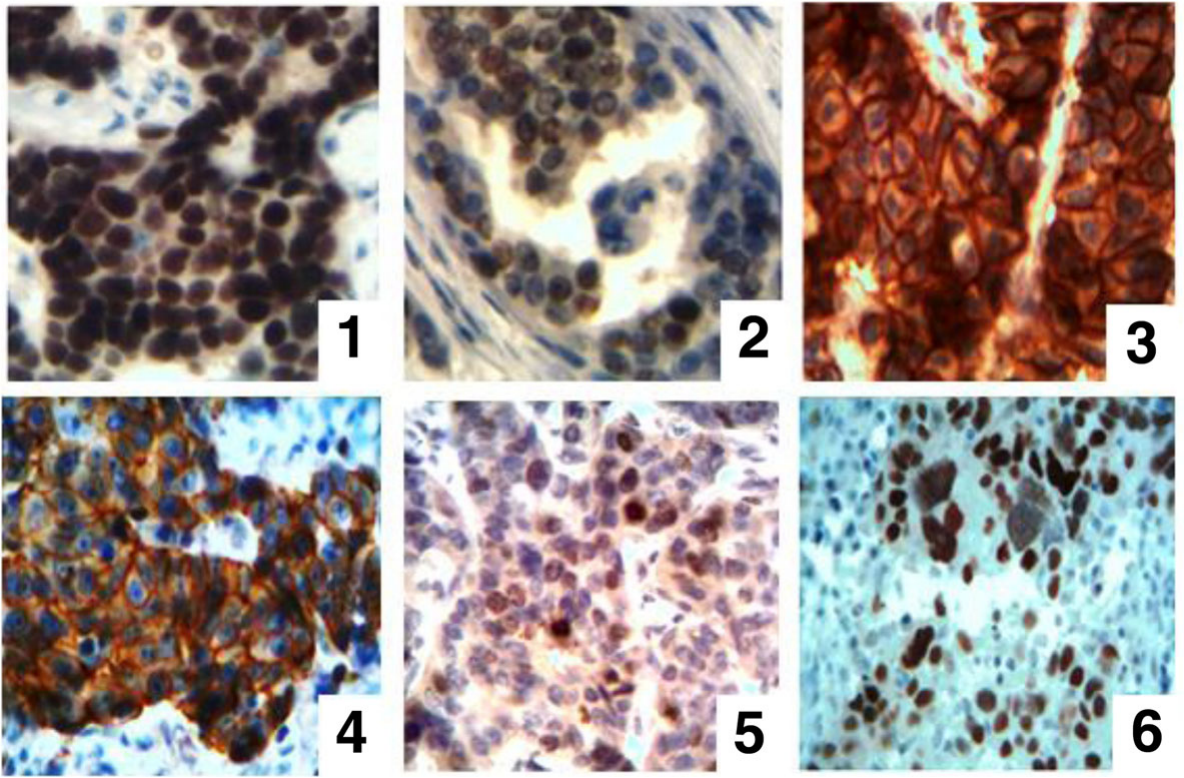
The immunohistochemical study of BC tissue. **1** – ER, **2** – PR, **3** – HER2/neu, **4** – E-cadherin, **5** – Ki-67, **6** – p53. Magnification ×400

Immunohistochemical analysis has revealed different variants of receptors expression. 63% of tumors were ER-positive that was evaluated according to D.C.Allred recommendations. In 50% of cases we have revealed the presence of PR. Investigating the presence of HER2/neu receptors we have found that 12 samples were receptor positive. Studying the expression of Ki-67 receptor we have found that IDC tissue in 87.5% of cases possessed varying degree of proliferative activity. The presence of mutated proapoptotic protein p53 (mt p53) has been found in 37.5% of samples of BC (the possibility to identify wt p53 protein was absent due to the short time of its existence and its below a threshold level – this number is impossible to detect by immunohistochemical studies). The examples of receptor-positive variants of BC tissues are shown in Fig. 3. The results of statistical data processing of immunohistochemical studies have found that in our research the correlative connections were as follows: the positive correlative connections between ER and PR (*r* = 0.85, *p* < 0.01), p53 and Ki-67 (*r* = 0.55, p < 0.01); the negative correlative connections between ER and HER2/neu (*r* = −0.56, p < 0.01), PR and HER2/neu (*r* = −0.58, p < 0.01), ER and Ki-67 (*r* = −0.37, *p* < 0.05), PR and Ki-67 (*r* = −0.33, p < 0.05).

**Fig. 4:**
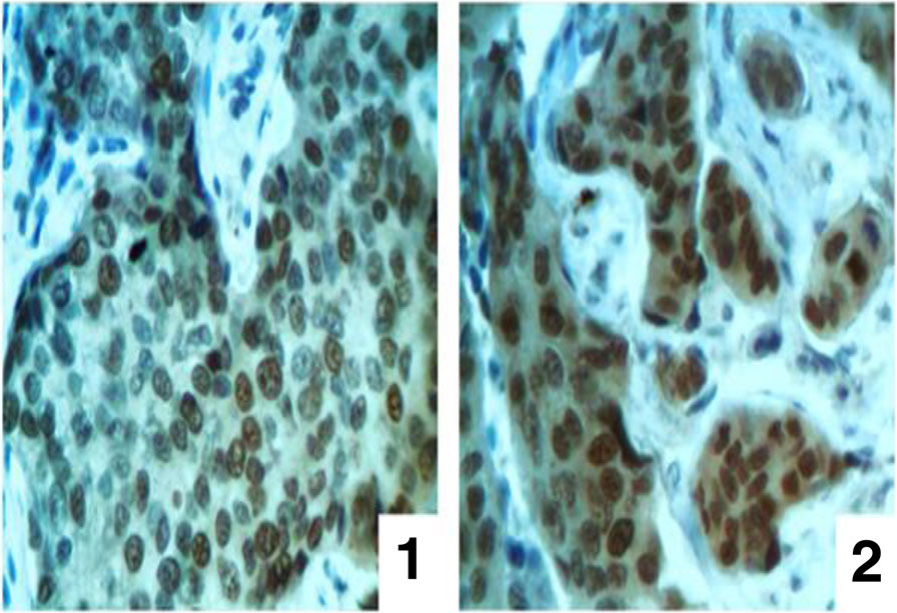
The immunohistochemical study of MGMT. **1** – IDC tissue with nuclear localization of the enzyme, **2** – IDC tissue with nuclear and cytoplasmic localization of the enzyme. Magnification ×400

The patterns of MGMT expression, according to the recommendations of the manufacturer of the antibodies, were cytoplasm and nucleus (Fig. 4). In our studies it has been received more pronounced nuclear localization of MGMT. This confirms the basic function of MGMT, which carries an alkyl residue (methyl-, ethyl-, n-propil- and others) from O^6^ position of guanine on its 145th active site which respectively is localized in the nucleus of “cancer” cells. We have not established the cases in which pattern of expression was only cytoplasm. During research we have revealed the following results: 17 cases (42.5%) of IDC were MGMT-positive and 23 samples (57.5%) – MGMT-negative. Cytoplasmic expression of MGMT has been found in almost 30% of BC cases. Investigating the combination of MGMT and ER expression we have revealed an interesting pattern – during the increase of MGMT expression the level of steroid hormone receptors decreased (*r* = -0.5, *p* < 0.01).

According to the results of spectroscopy, the range of DNA fluctuations is located within 400–4000сm^-1^. We conventionally divided obtained infrared DNA spectra into three areas: the first area corresponded to the spectrum of nitrogenous bases fluctuations (4000-2000 cm^-1^), the second – the fluctuations of deoxyribose CH_2_-groups (1700-1500 cm^-1^) and the third – the fluctuations of the deoxyribose and phosphate groups in the skeleton of DNA molecules (1300-1000 cm^-1^). The spectra were divided into weak ≤5%, average – 5-20% and strong – ≥20%, depending on the intensity of infrared absorption. The attention was also paid to the level of manifestation of vibrational spectrum and its length. The main purpose of the study was to identify the DNA methylation level by the indicators of the deviation of absorptive waves in 2850 cm^-1^-3050 cm^-1^ range, corresponding to methyl (-CH_3_) group fluctuations.

**Fig. 5:**
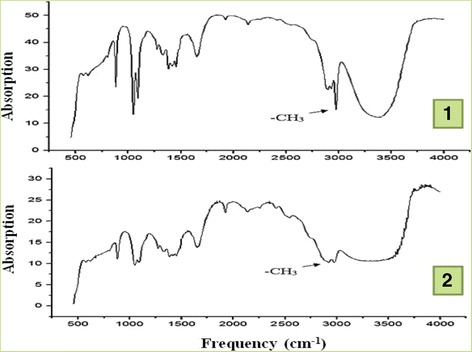
Infrared spectrum of DNA. **1** – spectrum with one peak of methyl group fluctuation, **2** – spectrum with the splitting of infrared fluctuations of -CH3 groups

The results have shown that the spectrum of methyl group fluctuation was in the range from 2893 cm^-1^ to 3048 cm^-1^ and it presented in 92.5% (37 cases) as one peak, and in 7.5% (3 cases) there was the splitting of infrared fluctuations of -CH_3_ groups (Fig. 5). The peak of -CH_3_ fluctuations was in the range from 2920 cm^-1^ to 2991 cm^-1^. Its intensity was in the range from 76.15% to 98.72% (average 89.71%), it being a strong peak. Therefore as -CH_3_ fluctuations is in the range of fluctuations of nitrogenous bases (4000-2000 cm^-1^), we can assume that the process of pathological methylation takes place in the adenine, guanine and cytosine or thymine additionally adds a methyl group. We found moderate positive correlative relationship between the level of MGMT expression and DNA methylation levels (*r* = 0.53, *p* < 0.01), between the level of infrared spectrum absorption and level of p53 expression (*r* = 0.32, *p* < 0.01). We observed the tendency of decreasing the expression of ER and PR with the growth of abnormal DNA methylation.

Comparing the results of the chemical composition of IDC tissue with results of immunohistochemical investigations we have got the following results: the growth of HM in neoplastic tissue is accompanied with the increase of HER2/neu (*r* = 0.36, *p* < 0.05), p53 (r = 0.53, p < 0.01), Ki-67 (*r* = 0.47, *p* < 0.01), MGMT (*r* = 0.34, p < 0.05) expression and decrease of ER (*r* = -0.71, p < 0.01) and PR (*r* = -0.66, p < 0.01) expression. The growth of pathological DNA methylation is also accompanied with the increasing amount of HM in tumor tissue (*r* = 0.41, p < 0.01).

General information concerning the presence of correlation connections between different BC indicators are presented in Table 3.

**Table 3:**
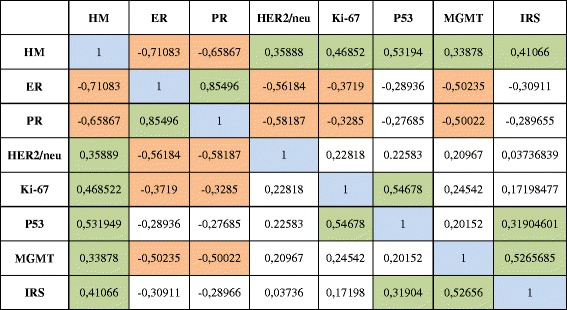
Factor analysis of IDC indicators

*IRS* infra red spectrum. **Orange color** – a negative correlation between HM and ER, HM and PR, ER and HER2/neu, ER and Ki-67, ER and MGMT, PR and HER2/neu, PR and Ki-67, PR and MGMT. **Green color** - a positive correlation between HM and HER2/neu, HM and Ki-67, HM and P53, HM and MGMT, HM and IRS, P53 and Ki-67, IRS and P53, IRS and MGMT. **Blue color** - intersection of the same indicators

It should be noted that all the molecular-genetic indicators of IDC progression authentically increased while reducing of tumor differentiation degree.

## Discussion

Our present is characterized by the growing influence of environmental factors upon human health. Without a doubt, HM are vital micro elements which are included in the vital functions of all body cells. But their function is changing when they arrive in excessive quantities to the intracellular environment, showing their toxic functions. According to the results of spectrophotometry IDC tissue contains in its composition different amount of HM. The reason is that the cancer cells at the expense of disorders of endo-exocytosis of chemicals accumulate HM, in accordance with the growth of their number in the blood vessels (due to the increased receipts of HM to the body). We can explain the increase of heavy elements concentration in the neoplastic tissue with the progression of carcinogenesis at the expense of violation of regulatory mechanisms of HM assimilation and utilization by tumor cells. Increasing level of cell anaplasia leads to blocking the synthesis of enzymes involved in maintaining of intracellular homeostasis. On the other hand, these microelements stimulate this imbalance by supporting of HM ominous impact on the course of malignancy.

HM accumulation impacts on cell phenotype. This process is realized through a block of steroid receptors that reduces the sensitivity of cancer cells to anti-hormone therapy. This is due to DNA methylation and increases the amount of MGMT (see below), the initiation of inflammation and inhibition of ESR genes transcription [26], depletion of ER and progression of phenotypic simplification [27].

On the other hand, heavy microelements activate antiapoptotic function and proliferative potential of cells by the synthesis of mt p53 [10]. As shown by our study MGMT protein is no exception. Its quantity grows with increasing HM intracellular content. We explain the protein growth level through different effects of heavy metals on MGMT synthesis. In our opinion one of the possible points in this chain is their impact on cell DNA. In this case HM might lead to the increment of methyl group accession to the DNA molecule. This fact is not accidental, because the HM are involved in many intracellular processes, including the transfer of electrons and active groups (-CH_3_, -O, -S) [28]. We have found a positive correlative relationship between DNA methylation level and the amount of HM in the tumor tissue during our study.

**Scheme 1:**
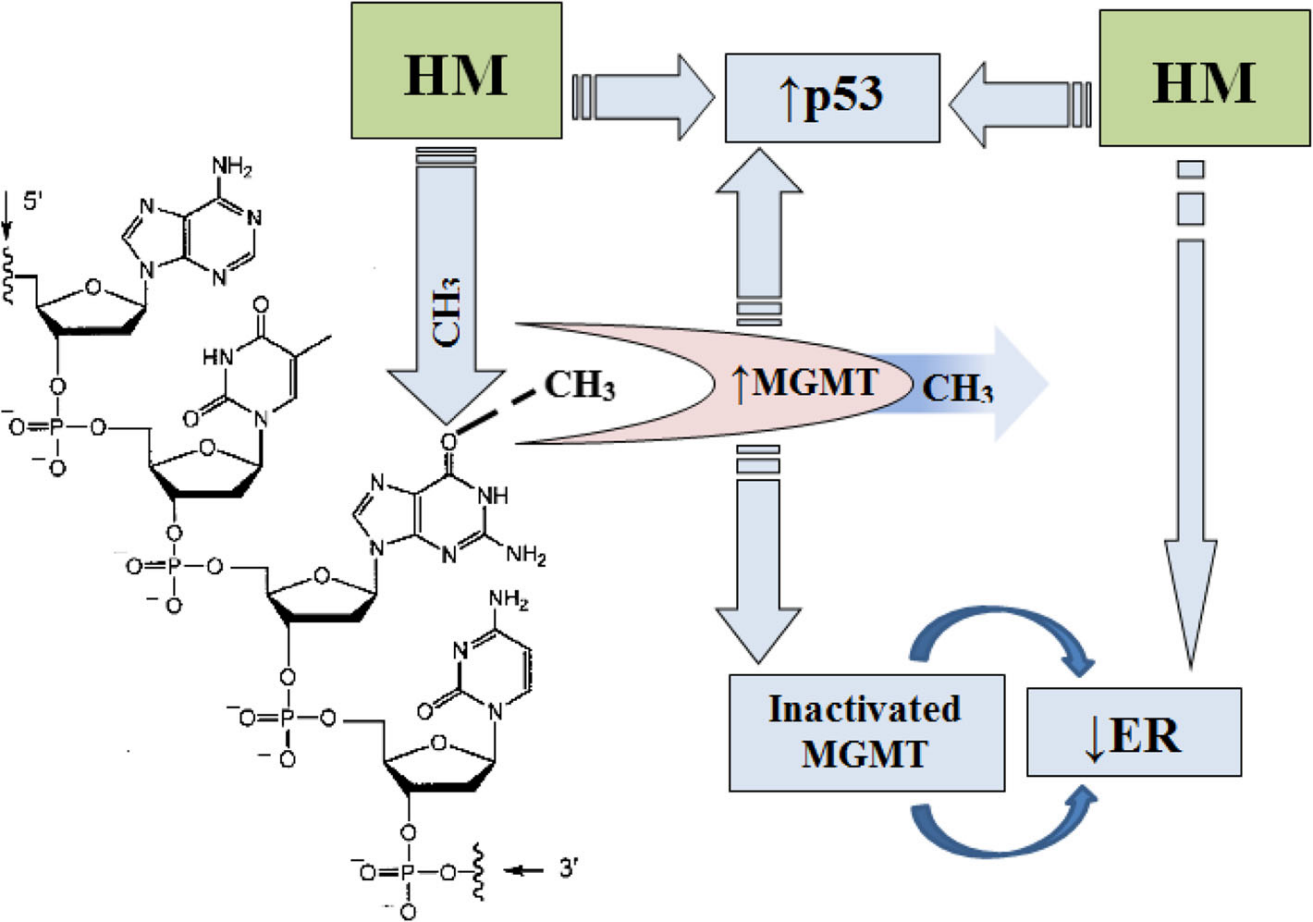
The pathological DNA methylation and its effect on the receptor profile of tumor tissue

The general scheme of pathological DNA methylation and MGMT synthesis is as follows (Scheme 1). An exogenous revenue of heavy metal salts into the body leads to their deposition in tumor tissue [29]. On the one hand they block the reparative systems [7], on the other hand they can stimulate the transfer of -CH_3_ groups [28]. As a result methyl groups are joined to thymine (only thymine (C_5_H_6_N_2_O_2_) has in its composition in norm -CH_3_ group) and -CH3 groups to other nitrogen bases. The excessive abnormal DNA methylation can stimulate the reparative system activation, which is represented by MGMT protein, which permanently removes attached -CH_3_ group from nitrogenous bases [20].

Inactivated MGMT enters into relationship with receptors for steroid hormones inhibiting their function [21]. This fact is confirmed by the presence of a negative correlative connection between the MGMT expression and steroid receptors. The lack of relationship between the MGMT, p53 and Ki-67 expression indicates separate enzyme impact on the course of the malignant process that does not depend on the presence of other disorders in the pathogenesis [23]. Instead, the level of p53 protein expression increased together with increasing of the level of DNA methylation. This indicates the impact of the methylation on cancer cells pro-apoptotic function [17].

The relationship between the expression of ER and PR, ER and HER2/neu, PR and HER2/neu, p53 and Ki-67, ER and Ki-67, PR and Ki-67 indicate the presence of close cooperation between the various links of IDC progression [10, 30].

## Conclusions

Heavy metals are accumulated in breast cancer tissue. The total amount of them range from 51.21 × 10^-3^ to 84.86 × 10^-3^ μg/kg (average 72.44 × 10^-3^ μg/kg) in this tumor. They are accumulated more in the parenchymal component of tumor tissue. We have established the relationship between the heavy metals accumulation in the foci of neoplasia and degree of breast cancer malignancy.

DNA of tumor tissue has a different level of methylation which changes with the amount of heavy metals in cancer cells (a positive correlation between the amount of heavy metals in the neoplastic tissue and DNA methylation level has been established – *r* = 0.41, *p* < 0.05). This is displayed on the synthesis of prognostically important receptors (MGMT, p53) in neoplastic tissue. Inactivated MGMT can enter into relationship with receptors for steroid hormones inhibiting their function.

The growth of heavy metals in neoplastic tissue is accompanied with the increase of HER2/neu, p53, Ki-67, MGMT expression and decrease of ER and PR expression. The relationship between the expression of ER and PR, ER and HER2/neu, PR and HER2/neu, p53 and Ki-67, ER and Ki-67, PR and Ki-67 indicate the presence of close cooperation between various links of invasive ductal carcinoma progression.

Heavy metals through different pathogenetic links stimulate the progression of breast cancer and reduce its sensitivity to treatment.

## Abbreviations

BC: Breast cancer
ER: Estrogen receptor
HER2/neu: Human epidermal growth factor receptor 2
HM: Heavy metals
IDC: Invasive ductal carcinoma
MGMT: O6-methyguanine-DNA methyltransferase
mt p53: Mutant type p53
PR: Progesterone receptor
wt p53: Wild type p53
